# A 40-Year-Old Woman Who Developed Jaundice during Therapy for Thyrotoxicosis

**DOI:** 10.1371/journal.pmed.0030012

**Published:** 2006-01-31

**Authors:** Chukwuma Ogbonna Ekpebegh, Naomi Sharlene Levitt

## Abstract

Ekpebegh and Levitt discuss the differential diagnosis of jaundice in patients with thyrotoxicosis.

## DESCRIPTION of CASE

A 40-year-old woman presented to a tertiary-care facility on the 11th of October 2004, having been referred from a private-health clinic for continued treatment of thyrotoxicosis. The referring doctor had started her on carbimazole, 60 mg daily 11 months earlier. She discontinued treatment after three months for financial reasons. She was referred to the state tertiary facility because the fees there are subsidized.

At presentation on the 11th of October 2004, she had no overt symptoms of hyperthyroidism. Physical examination revealed a well-looking woman with a soft, smooth, nontender moderately sized diffuse thyromegaly with no bruit. Her palms were warm and moist with palmar erythema. There was a mild tremor of the outstretched fingers. There were no obvious eye signs of thyrotoxicosis or Graves disease. The patient's heart rate was 87 beats per minute and regular, with a blood pressure of 134/70 mm Hg. There were no features of cardiac decompensation or proximal myopathy.

Her thyroid function test results and therapeutic interventions are as shown in Table 1. Thyroid antimicrosomal and antithyroglobulin antibodies were positive with titres of 1:400 and 1:40, respectively ( Table 1).

**Table 1:**
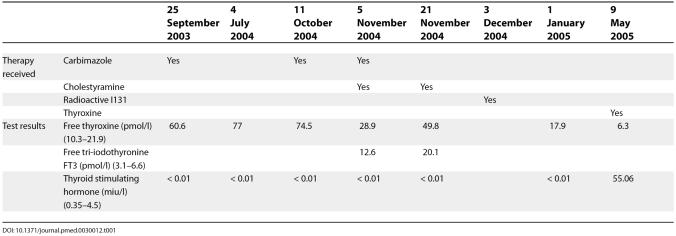
Thyroid Function Test Results and Dates Performed with Therapy Received

### What Was the Diagnosis and Treatment?

A diagnosis of thyrotoxicosis due to Graves disease was made on the basis of smooth diffuse thyromegaly and positive markers of thyroid autoimmunity. As the patient had not received adequate medical therapy, carbimazole was reintroduced at 40 mg daily. She was counselled that adequate medical treatment would require daily compliance with the prescribed medication for a period of 12–18 months. She was hopeful about complying with this, as the medical fees were affordable.

However, she re-presented three weeks later with progressively deepening jaundice, pruritus, and passage of clay-coloured stools. There was no previous history of jaundice, blood transfusions, intravenous drug abuse, anaesthesia, recent history of travel, or animal exposure. Her son, however, had recently become jaundiced. She did not consume alcohol, but did receive a two-monthly injectable contraceptive progestagen (Nuristerate) for the four years preceding presentation.

She was markedly jaundiced on examination. There were no peripheral stigmata of chronic liver disease. There was no right hypochondrial tenderness, hepatomegaly, features of hepatic encephalopathy, or cardiac decompensation.

### What Is the Differential Diagnosis of Jaundice in a Patient with Thyrotoxicosis?

Several causes of jaundice should be entertained in this patient. The aetiology may be multifactorial. The differential diagnosis of this patient's jaundice can be divided into (1) thyrotoxicosis per se and conditions associated with autoimmune thyroid disease, including thionamide therapy, hepatic congestion from thyrotoxic heart failure, hepatic necrosis from systemic embolisation caused by atrial fibrillation, autoimmune hepatitis, and primary biliary cirrhosis, which may be associated with Graves disease, and (2) causes of jaundice unrelated to thyrotoxicosis, such as viral hepatitis, sepsis, cholangitis, alcohol abuse, and medication such as hormonal contraceptives, acetaminophen, isoniazid, rifampicin, and herbal remedies.

The pattern of liver function test abnormalities may suggest an aetiology for the jaundice. While hepatocellular damage is classically characterised by elevated serum transaminase levels with minimal increase in serum alkaline phosphatase (hepatocellular pattern), the converse is the case with obstructive causes of jaundice (obstructive pattern). However, many conditions that cause jaundice give a mixed hepatocellular/obstructive pattern.

The recent onset of jaundice in her son suggested the possibility of viral hepatitis. She was subsequently managed as an in-patient from 5 November 2004–29 November 2004.

### Which Investigations Were Performed?

Haemoglobin and white blood cell count were normal at 12.1 g/dl and 10,400/cm ^3^, respectively. Serum urea, electrolytes, and creatinine were normal.

Her serology was negative for hepatitis A anti-IgM antibody, hepatitis B surface antigen, hepatitis B core anti-IgM, hepatitis B surface anti-IgM, hepatitis B antigen, and hepatitis C virus anti-IgM. She was, however, positive for Hepatitis C anti-IgG, indicating prior exposure to hepatitis C. Her serial liver function test results are shown in Table 2.

**Table 2:**
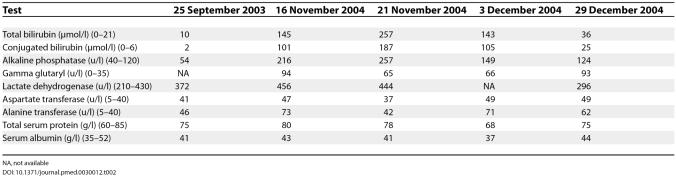
Serial Liver Function Test Results

NA, not available

Her antinuclear antibody was positive at a titre of 1:160. Anti-liver-kidney microsomal and antimitochondrial antibodies were negative. The abdominal ultrasound demonstrated a normal sized liver with no focal lesions. The bile ducts, pancreas, kidney, and spleen were all reported as normal.

In view of the negative hepatitis serology and the possibility of autoimmune hepatitis, a liver biopsy was undertaken once contraindications to the procedure (such as derangements in coagulation profile) had been excluded. One may, however, argue that a conservative approach could have been taken. In other words, rather than performing a liver biopsy, we could have opted to observe for the expected improvement in liver function test abnormalities and jaundice on discontinuation of the carbimazole therapy. The decision to perform a liver biopsy in this patient was based on the need to exclude autoimmune hepatitis, given the fact that she already had elevated liver enzymes before starting carbimazole therapy and given the raised serum titres of antinuclear antibodies. Autoimmune hepatitis may have been an additional cause of the patient's jaundice that would have needed treatment in its own right.

Liver biopsy is not without risks, which include haemoperitoneum, biliary peritonitis, sepsis, and pneumothorax. Precautionary measures are needed, such as knowing the patient's coagulation status, having standby cross-matched blood, and strict asepsis and periodic monitoring of haemodynamic status after the procedure. Ultrasound guidance may be necessary in the following conditions: obesity, ascites, reduced liver span, and focal hepatic lesions. Our patient had a blind liver biopsy after initial ultrasound scan had shown a normal liver size with no focal lesions. The biopsy tract was, however, plugged with gelatin foam to prevent bleeding. The histological finding was of striking cholestasis and lobular inflammation, which was predominantly lymphocytic. There were few neutrophils and eosinophils with mild portal inflammation. There were, however, no plasma cells as would be expected in autoimmune hepatitis.

### What Was the Cause of Jaundice in This Patient?

Although her son proved to have active hepatitis A infection, this was excluded in our patient by negative serology. The probable cause of jaundice would appear to be carbimazole since it developed three weeks after it was recommenced. The presence of eosinophils on liver histology was suggestive of an allergic aetiology for the hepatobiliary damage. Carbimazole-induced hepatic damage is thought to be allergic in aetiology and is typically predominantly cholestatic, as depicted in this patient ( Table 2).

Thionamide-induced hepatobiliary damage usually occurs within three months of therapy and may occur despite previous uneventful exposure to the drug. A contributory effect from hormonal contraceptive use cannot be excluded. The liver enzyme tests done by the referring facility as of 25 September 2003 (i.e., even before she was started on carbimazole therapy) were already mildly deranged ( Table 2).

### How Did the Patient's Investigations Affect Her Management?

Carbimazole therapy was immediately discontinued, and the patient was advised about alternative nonhormonal methods of contraception. She was started on cholestyramine, 12 g daily in divided doses, and atenolol (a beta-blocker), 50 mg daily. A nonselective beta-blocker such as propranolol is preferable when choosing a beta-blocker for treating thyrotoxicosis. High doses should be used as the thyrotoxic state potentiates the metabolic clearance of beta-blockers. Propranolol, in addition to nonselective beta-blockade, has the benefit of inhibiting the peripheral conversion of thyroxine to the more potent triiodothyronine. While cholestyramine interferes with the enterohepatic circulation of thyroid hormones, atenolol attenuates the peripheral effects of circulating thyroid hormones. Radioactive iodine therapy was subsequently administered once pregnancy had been excluded.

### How Did the Patient Respond?

The jaundice resolved over a period of three months from presentation. Hypothyroidism was diagnosed five months after radioactive iodine treatment, and thyroxine, 50 mcg daily, was started.

## DISCUSSION

Thyroid dysfunction may perturb liver function, and the liver modulates thyroid hormone metabolism. A variety of systemic diseases and drugs may affect both organs [ 1, 2].

### Thyrotoxicosis and Hepatobiliary Injury

Abnormal liver biochemical test results have been reported in hyperthyroid patients before and after antithyroid therapy. Gurlek et al. [ 3] showed that 60.5% of 43 patients with hyperthyroidism had at least one liver abnormality at diagnosis. Hepatic damage occurring from thyrotoxicosis per se has been ascribed to ischaemic injury resulting from a relative decrease in blood flow despite increased metabolic activity of the liver [ 1]. But bear in mind that raised alkaline phosphatase may not be of liver origin but rather from bone, indicating an osteoblastic response to thyroid hormone-induced bone resorption.

The nature of hepatic injury caused by thionamides is dependent on the specific drug. While carbimazole and its active metabolite methimazole typically cause cholestasis, propylthiouracil (PTU) is notable for causing hepatocellular injury [ 4–7]. Thionamide-induced liver damage is an idiosyncratic reaction that can develop at any time, but usually occurs within the first three months of treatment. It occurs in about 1% of patients, with a predisposition for women younger than 30 years of age [ 1]. The mechanism of injury is thought to be based on an allergic host response [ 1, 4, 8].

Our patient had a predominantly cholestatic hepatitis, which is consistent with cases of carbimazole- and methimazole-induced hepatic damage reported in the literature [ 9–11]. Thionamide therapy may be an additional insult to the liver. Khoo Ai-Leng et al. [ 12] reported a case of fatal hepatic failure in a patient on carbimazole and bupropion, while Enghofer et al. [ 13] reported fulminant hepatitis A infection in a hyperthyroid patient treated with carbimazole. Therefore, we suggest that it might be prudent to exclude additional risk factors for liver injury in patients presenting with thionamide-associated cholestasis.

### Therapeutic Options in Our Patient

Substitution of one thionamide for the other has been suggested as a treatment approach for thionamide-induced hepatotoxicity. Indeed, Waseem et al. [ 14] reported a successful outcome when a patient with PTU-induced hepatitis was treated with a combination of methimazole and lithium. We, however, did not consider this option in our patient, as PTU is not readily available in South Africa and it is potentially hepatotoxic. After initial therapy with cholestyramine and atenolol, the patient received radioactive iodine therapy. This was not associated with additional morbidity. Her outcome was in keeping with the review of a meta-analysis of reports of patients with PTU-associated hepatotoxicity, which showed favourable outcomes despite most patients receiving radioactive iodine before hepatic function test abnormalities had resolved [ 8]. While thyroidectomy is a preferred modality of management in many centres, we rarely use it. Hypothyroidism was diagnosed five months after radioactive iodine therapy, and thyroxine therapy was promptly started. Hypothyroidism may, however, develop many years after radioactive iodine therapy. This underscores the need for life-long follow-up after radioactive iodine therapy, as hypothyroidism does cause considerable morbidity and can trigger or exacerbate thyroid eye disease.

**Key Learning Points**

- Jaundice as a complication of thionamide treatment of hyperthyroidism is rare.
- This complication cannot be predicted by deranged liver enzymes at presentation, but typically occurs within three months of therapy.
- It can be fatal, particularly when there are additional hepatotoxic factors.
- The subsequent management of hyperthyroidism should ideally be with proven alternative methods without a hepatotoxic potential.

## Abbreviation

PTU: propylthiouracil
